# Effects of 3D Virtual Reality on Postural Control in Young Adults: Clinical and Practical Implications

**DOI:** 10.3390/clinpract16020040

**Published:** 2026-02-13

**Authors:** Gustavo Christofoletti, Gabriela Maria da Silva Béé, Otávio Reginato, Nathalia Oliveira Rodrigues, Sidineia Silva Pinheiro Cavalcante Franco, Ana Beatriz Gomes de Souza Pegorare

**Affiliations:** Institute of Health, School of Medicine, Federal University of Mato Grosso do Sul, UFMS, Campo Grande 79060-900, Brazil; gabriela.maria@ufms.br (G.M.d.S.B.); otavio.reginato@ufms.br (O.R.); nathaliar861@gmail.com (N.O.R.); sidineiapinheiro27@gmail.com (S.S.P.C.F.); ana.pegorare@ufms.br (A.B.G.d.S.P.)

**Keywords:** virtual reality, young adult, postural balance, evidence-based practice

## Abstract

**Background:** Previous studies have demonstrated the benefits of virtual reality (VR) as an intervention tool guided by specialists. However, little is known about whether VR may pose risks in uncontrolled environments. Considering its implications for clinics and practice, this study aimed to assess the potential risks of a 3D VR simulation on postural control in young adults. **Methods:** Seventy-nine community-dwelling young adults completed a VR program using a head-mounted display that simulated a 3D roller-coaster ride while standing. Postural control was assessed using a force platform measuring frontal and lateral sway, center-of-pressure sway area, and frontal and lateral imbalance speed. The assessments were conducted with and without VR. Statistical analyses were performed using paired comparisons. Significance was set at 5%. Effect sizes (ESs) are reported. **Results:** Engaging in a VR roller-coaster simulation increased the participants’ imbalance in terms of frontal sway (*p* = 0.001; ES = 0.919), center-of-pressure sway area (*p* = 0.001; ES = 0.849), frontal imbalance speed (*p* = 0.001; ES = 0.910), and lateral imbalance speed (*p* = 0.001; ES = 0.663). No significant difference was observed in the lateral sway (*p* = 0.383). During VR exposure, 25% of the participants showed a clinically significant increase in postural instability. Despite having normal baseline parameters, participants with higher postural instability showed greater deterioration in postural control during VR exposure than those with lower postural instability. **Conclusions:** A 3D VR simulation affected several measures of postural control in community-dwelling young adults. Precautions should be taken when engaging in VR without appropriate specialist supervision.

## 1. Introduction

Virtual reality (VR) is a rapidly evolving technological innovation that enables the creation and representation of immersive three-dimensional environments. Originally developed for entertainment purposes (such as video games, simulators, and interactive media), VR has progressively expanded into multiple domains, including medicine, architecture, biology, astronomy, and education [1,2]. Advances in computing processors, motion tracking, and display technologies have contributed to the growing accessibility and widespread adoption of VR across both clinical and non-clinical contexts.

A defining characteristic of VR is its capacity to create immersive environments that allow users to interact with diverse scenarios. This interaction is typically achieved through motion sensors that capture body movements and translate them into the virtual environment, as well as through head-mounted displays that visually isolate the users from the physical world and replace it with a digitally constructed reality [3,4]. By providing highly salient visual input and multisensory stimulation, VR systems fundamentally modify how visual, vestibular, and proprioceptive information is integrated to support postural control.

The application of VR has demonstrated substantial benefits in a variety of settings. In medicine, VR has been widely used as a training tool, enabling surgeons and healthcare professionals to practice complex procedures in a safe and controlled environment without exposing patients to risk [5]. In educational contexts, VR offers innovative approaches for teaching and learning, where students can visualize, manipulate, and explore three-dimensional structures [6]. Furthermore, VR-based interventions have shown promising results in the management of mental health conditions such as anxiety [7] and depression [8], as well as in neurorehabilitation programs targeting balance impairments [9] and movement disorders such as ataxia [10]. Collectively, these findings highlight the broad potential of VR as a therapeutic, educational, and training tool.

Despite the clinical benefits, a substantial proportion of VR practice remains concentrated on entertainment applications, especially video games and immersive simulations. Young adults represent a major segment of VR users who frequently engage with these technologies in leisure contexts. In contrast to clinical applications, entertainment-based VR is often used without professional supervision or standardized safety guidelines. Notably, studies examining the potential risks of VR practice in young individuals in the absence of direct specialist supervision remain limited [11,12].

The potential effects associated with inappropriate use of digital technologies have been previously documented in the literature. Similarly to VR, smartphone use while performing a concurrent task represents a classic dual-task scenario that has been consistently associated with impaired motor performance, increased attentional demand and an elevated risk of accidents [12,13,14,15]. These findings underscore how competition for cognitive and sensory resources compromises motor control and safety. Within this dual-task framework, competition for limited attentional resources is considered a key mechanism underlying the deterioration of motor control during simultaneous cognitive engagement.

Building on evidence from dual-task research, smartphone use has been shown to interfere with motor activity primarily by dividing attention between competing tasks and disrupting visual processing [16,17]. These mechanisms are particularly relevant when considering VR, which may impose even greater challenges on postural control due to its immersive visual nature. VR exposure not only increases cognitive and attentional load but also induces sensory conflict by presenting visually rich motion cues that may be incongruent with vestibular and proprioceptive inputs. Visual stimuli presented in VR may conflict with vestibular and proprioceptive inputs, leading to distortions in perception of distance, depth, and self-motion. Such sensory conflicts can compromise balance regulation, increase postural sway, and elevate the risk of missteps, accidents, and falls [18,19,20].

From a sensory perspective, the central nervous system responds to these conflicts through sensory reweighting processes, often increasing reliance on visual input while down-weighting somatosensory and vestibular information. Although adaptive in certain contexts, excessive visual dependence may compromise postural stability, even in young and healthy individuals [4].

Because evidence regarding the risks of VR use remains limited, its implications for clinical and applied practical settings warrant careful consideration. Taken together, these mechanisms support a conceptual framework in which VR-induced visual immersion increases attentional demand and sensory conflict, leading to altered sensory reweighting and postural control strategies.

Given its immersive and sensory-altering nature, VR may impose greater demand on attentional allocation and sensory integration than other digital technologies, potentially leading to clinically relevant balance and motor control impairments [15,16]. These considerations highlight the need for careful supervision, appropriate protocols, and risk awareness when implementing VR in clinical practice and in daily use [17].

As VR technologies become increasingly accessible and are being adopted in clinics and routine settings, understanding their potential risks is essential to ensure their safe implementation. Moreover, unsupervised use of VR in non-controlled environments may expose users to postural instability without adequate awareness of these risks. In this scenario, this study aimed to assess the effects of VR on postural control in community-dwelling young adults.

The following hypotheses were tested: H1: Exposure to an immersive 3D VR environment would significantly increase the center-of-pressure (CoP) sway area, indicating reduced postural stability under dynamic visual stimulation. H2: The destabilizing effects of VR exposure on postural control would be more pronounced in the anteroposterior direction than in the mediolateral direction, reflecting the predominantly anteroposterior optic-flow characteristics of the roller-coaster simulation.

The findings of this study may contribute to a better understanding of VR-related safety concerns and provide guidelines for its use in both clinical and practical settings.

## 2. Materials and Methods

This study included 79 community-dwelling young adults with a mean age of 21.5 ± 2.5 years. Data were collected at the outpatient clinic of the Federal University of Mato Grosso do Sul, Brazil. The study was conducted in accordance with the STROBE statement checklist, principles of the Declaration of Helsinki, and guidelines for Good Clinical Practice. The study protocol was approved by the Institutional Ethics Committee (approval number: 6.264.348). All participants provided written informed consent prior to the assessments, and the raw data are presented in Table S1.

Participants were eligible if they were young adults aged between 18 and 25 years, of either sex, and without any neurological or musculoskeletal disorders. Individuals were excluded if they reported a previous diagnosis of vestibular disorders, had undergone surgery within the previous six months, or were unable to attend the outpatient clinic for assessment. Prior exposure to virtual reality was assessed during screening, and only participants without previous experience using head-mounted VR systems were enrolled.

Participants were recruited directly by the research team through in-person invitations and announcements disseminated through social media platforms. Individuals who expressed interest were screened for eligibility. After providing informed consent, eligible participants completed a general questionnaire to collect demographic and anthropometric data, including sex, age, body mass, height, and body mass index (BMI).

**Figure 1:**
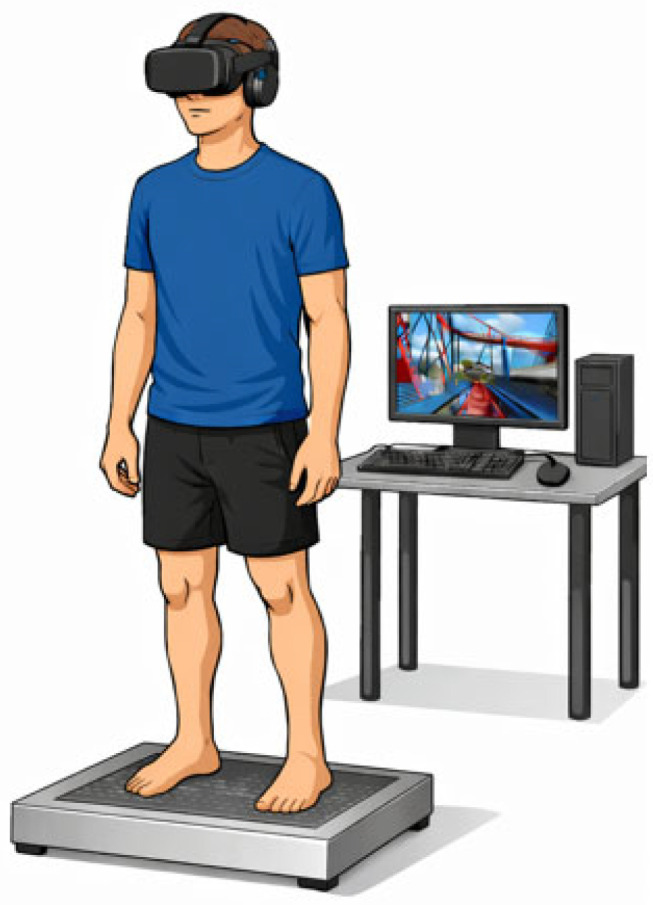
Three-dimensional virtual roller-coaster scene and the complete experimental setup.

ChatGPT (version 5.2) was used to generate Figure 1 at the authors’ request. Additionally, ChatGPT (version 5.2) was used for language refinement of the manuscript. The authors reviewed and edited all outputs to ensure scientific accuracy and clarity.

### 2.1. Sample Size, Blinding, and Randomization

Sample size estimation was performed using the G*Power software, version 3.1.9.7 for Windows (Heinrich-Heine-Universität Düsseldorf, Germany). The calculation was based on the effect size reported by Imaizumi et al. [4], who identified an effect size of 0.32 in young adults exposed to virtual reality. Assuming an effect size of 0.32, with a statistical power of 80% (β = 0.20) and an α error probability of 0.05, the analysis indicated that a minimum of 79 participants was required.

Since all participants performed postural assessments on a force platform under both VR and non-VR conditions, assessors were not blinded to the experimental conditions. Given the within-subject design of the study, participants served as their own controls, and group randomization was not applicable. However, to minimize potential order effects, the sequence of the experimental conditions was randomized. A rest period of 10 min was provided between conditions, during which participants remained seated and instructed to relax before the subsequent assessment.

### 2.2. Evaluative Assessments

All assessments were conducted at the Biomechanics Laboratory of the Outpatient Clinic of the Federal University of Mato Grosso do Sul. Static upright postural control was assessed using a force platform consisting of a 50 cm^2^ plate equipped with four load cells (BIOMEC 400_V4, EMG System do Brasil Ltda., São José dos Campos, Brazil).

The participants performed all assessments barefoot and were instructed to stand upright on the force platform for 180 s in each condition. The participants were asked to maintain a comfortable and natural standing posture without unnecessary movements. The following postural control variables were recorded: frontal and lateral sway, defined as the displacement of the body’s center relative to the base of support and expressed in centimeters (cm); CoP sway area, defined as the area covered by CoP displacement over time and calculated as an elliptical area expressed in square centimeters (cm^2^); and frontal and lateral sway velocity, defined as the speed of CoP displacement and expressed in centimeters per second (cm/s).

VR exposure was delivered using a 3D VR head-mounted display. The participants were assessed under two conditions, both with eyes open: a VR condition and a control condition without VR. During the VR condition, participants viewed a 3 min immersive 3D roller-coaster video while maintaining a stationary upright posture. In the control condition, the participants stood quietly for the same duration as without VR stimulation, maintaining a natural non-rigid posture. Participants who normally wore corrective lenses were permitted to wear them during testing, ensuring adequate visual acuity throughout the assessments. The VR video consisted of a roller-coaster simulation restricted to the frontal plane, minimizing lateral visual motion. Figure 1 illustrates the 3D roller-coaster virtual scene and the complete experimental setup.

### 2.3. Statistical Analysis

Statistical analyses were conducted using several steps. First, data distribution was assessed to verify compliance with parametric assumptions. Variables with normal distributions were summarized using means and standard deviations, whereas non-normally distributed variables were presented as medians and interquartile ranges. Second, to evaluate the effects of VR exposure on postural control, paired Student’s *t*-tests were applied for parametric data and Wilcoxon signed-rank tests were used for non-parametric data. Third, when statistically significant differences were identified, effect sizes were calculated and reported using Cohen’s d for parametric outcomes, and rank–biserial correlation coefficients for non-parametric outcomes. Quantile regression was applied to estimate VR-related effects at selected quantiles of the outcome distribution; no formal statistical comparisons between quantile-specific coefficients were performed. Significance was set at 5%.

## 3. Results

Ninety participants were initially recruited. Eleven individuals declined participation, citing difficulties in attending the outpatient clinic. A total of seventy-nine participants completed the study. Table 1 details individual characteristics, including age, sex, weight, height, and body mass index.

Table 2 presents the data for each variable measured on the force platform along with their respective analysis results. The frontal sway and lateral sway met parametric assumptions and are described using the mean and standard deviation. These variables were analyzed using Student’s *t*-test and Cohen’s d effect size. The remaining variables (CoP sway area, frontal speed, and lateral speed) did not meet parametric assumptions. These variables are described using the median and interquartile range and were compared using Wilcoxon W and rank–biserial correlation tests. No significant differences were observed between men and women for any postural control variable, either at baseline or during VR exposure (all *p* > 0.05).

**Figure 2:**
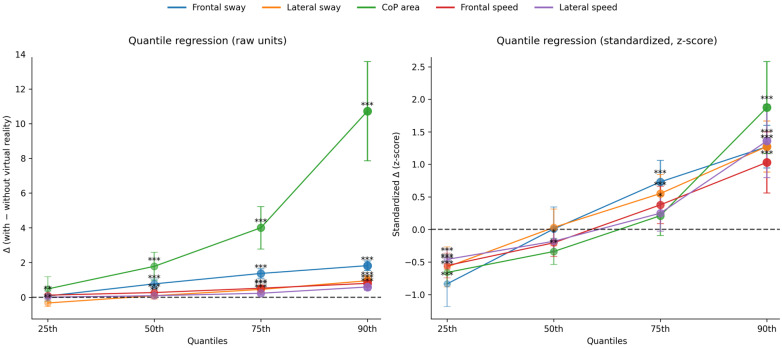
Quantile regression of postural control with and without standardization. Asterisks indicate levels of statistical significance (* *p* < 0.05; ** *p* < 0.01; *** *p* < 0.001).

Figure 2 shows the quantile regression analyses with standardized (z-score) and non-standardized (raw data) values. The analyses demonstrated that the impact of VR on postural control was not uniform across the distribution of the balance outcomes. For all variables, larger effects were observed in the upper quantiles, indicating that individuals with greater baseline instability experienced greater deterioration in postural control during VR exposure.

**Figure 3:**
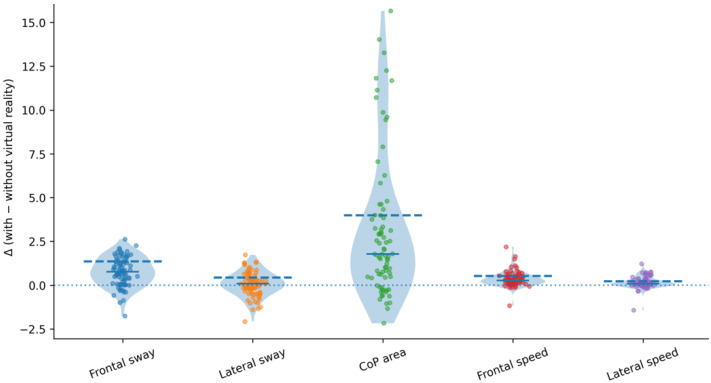
Distribution of postural control changes and clinically relevant thresholds during virtual reality exposure.

To estimate the proportion of individuals affected by VR, delta values were calculated for each variable. A clinically relevant increase in postural instability was defined as delta values that exceeded the 75th percentile of the corresponding distribution. Of the seventy-nine participants, 25% exhibited a clinically relevant increase in postural instability during VR exposure, indicating that a substantial subset of individuals may be particularly vulnerable to balance deterioration under immersive VR conditions. Figure 3 summarizes the distribution of the delta values across all domains measured in this study.

Although the primary outcomes of this study were objective postural measures, participants were verbally screened for symptoms of motion sickness, dizziness, or discomfort during and immediately after VR exposure. No participants reported motion sickness or balance-related discomfort.

## 4. Discussion

This study investigated the potential risks associated with VR exposure on postural control in community-dwelling young adults. The findings indicate that the use of a head-mounted display delivering a 3D roller-coaster simulation while standing induces a postural instability response, particularly affecting frontal sway, CoP sway area, and frontal and lateral sway speed. These results highlight that even short-term exposure to immersive VR content may challenge postural control strategies.

Young adults were targeted because they represent the population most frequently exposed to VR for entertainment, gaming, or education purposes. In contrast, middle-aged and older adults often demonstrate greater resistance to VR adoption. This resistance has been attributed to multiple factors, including difficulties in learning new technologies, challenges in handling VR equipment, and age-related changes in sensory integration that may compromise the interpretation of virtual stimuli [21,22,23]. Given these considerations, restricting the sample to young adults allowed the authors to investigate VR-related postural effects in a population known to have optimal sensory–motor responses.

The sample consisted predominantly of women (51 vs. 28 participants). As reported by Pastel et al. [24], men are generally less likely to volunteer for research, particularly in non-interventional or laboratory-based studies. Sociodemographic factors such as urban residence, employment status, and educational level may further limit the recruitment of more heterogeneous samples. In the present study, all participants were students residing in an urban area, which may restrict the generalizability of the findings to other sociodemographic contexts.

From a technological perspective, many contemporary applications, including games and emerging metaverse platforms, employ VR to promote social engagement for individuals of all ages [25,26,27,28]. However, metaverse environments typically involve interaction and complex cognitive demand, which activate executive functions and may independently influence motor behavior. This study employed a simplified VR task without social interaction to isolate the effects of visual immersion on postural control. This design was appropriated to reduce confounding effects related to cognitive demand, allowing clearer interpretation of the motor consequences of VR exposure.

At baseline, all participants exhibited postural parameters within normative ranges, consistent with previous reports in healthy young adults [29,30]. During VR exposure, however, significant alterations were observed in all postural variables, except for lateral sway. Although no formal adjustment for multiple comparisons was applied, all primary outcomes were predefined, and the main findings remained statistically significant even under conservative correction approaches, indicating a low likelihood of Type I statistical errors.

The absence of a significant change in lateral sway likely reflects the characteristics of the visual stimulus, which was primarily confined to the sagittal plane and required vertical head movements without corresponding lateral visual feedback. This interpretation is consistent with prior evidence demonstrating that the directionality of visual flow strongly influences the plane-specific organization of postural responses [31]. While this controlled design allowed the isolation of anteroposterior visual perturbations, it does not fully represent the complexity of recreational VR environments, which frequently involve multidirectional visual flow and rotational components.

Quantile regression analyses were included in this study to provide insight into inter-individual variability in postural responses to VR exposure. The analyses demonstrated that participants located in higher quantiles of baseline postural variability (although still within normative limits) exhibited greater VR-induced deterioration in postural control compared with those in lower quantiles. These findings indicate that VR-related instability is not uniformly distributed across individuals and highlight the presence of statistical heterogeneity in postural responses within a healthy young adult population.

Importantly, these quantile-specific effects should not be interpreted as evidence of clinically meaningful vulnerability or fall-risk stratification, as no clinical thresholds or risk classifications were applied. Rather, the results suggest that subtle pre-existing differences in postural control may influence sensitivity to immersive visual stimulation. From a clinical perspective, this observation underscores the potential relevance of baseline postural characteristics for understanding individual variability in VR responses.

Complementing the quantile results, delta analyses further clarified the magnitude of the VR-induced changes. The CoP sway area demonstrated the largest delta between VR and no-VR conditions. Unlike directional sway measures, CoP area captures the overall spatial dispersion of postural adjustments, integrating both anteroposterior and mediolateral components [32]. The pronounced increase in CoP area during VR exposure may reflect a generalized destabilization arising from sensory conflict and visual dominance, rather than isolated directional impairments. This finding supports the sensitivity of CoP area as a key indicator of VR-induced postural instability.

Although the absolute increases in frontal sway, CoP area, and sway speed were relatively modest, twenty-five percent of the participants exhibited a relevant increase in postural instability. This subgroup-level response would not be captured by mean comparisons alone and would reinforce the value of combining distribution-based (quantile) and change-based (delta) approaches. In community-dwelling young adults, such changes may not translate to immediate functional consequences. However, in populations with reduced postural responses, similar deltas could increase fall risks.

All statistically significant findings were accompanied by strong effect sizes and adequate statistical power, supporting the robustness of the results. These methodological strengths enhance confidence that the observed effects reflect true VR-induced alterations in postural control rather than random variability or sampling bias.

This study has some limitations that should be acknowledged. First, the findings are limited to healthy young adults and cannot be generalized to older or clinical populations. Age- and sex-related differences in postural control strategies, neuromuscular function, and sensory integration have been reported in the literature and may influence responses to immersive VR exposure. Second, the VR stimulus consisted of a single, passive roller-coaster simulation with motion predominantly restricted to the sagittal plane. Although this controlled design was intentionally chosen to reduce cognitive and motor confounding and to isolate the effects of immersive visual input on postural control, it limits the ecological validity of the task. Most contemporary VR applications are interactive, multidirectional, and cognitively demanding. Consequently, the present findings should not be generalized to VR use broadly and may underestimate the magnitude of postural instability associated with real-world VR exposure. Future research should examine more advanced VR systems, including those that incorporate multidirectional visual flow, active locomotion, and social interaction, to determine whether these features exacerbate postural instability. Such investigations are essential to establish evidence-based guidelines for the safe implementation of VR in both recreational and clinical settings.

## 5. Conclusions

Community-dwelling young adults exposed to a head-mounted display delivering a 3D roller-coaster simulation exhibited increased frontal sway, CoP sway area, and frontal and lateral sway speed. These alterations indicate a potential increase in fall risk when VR is performed in the upright standing position.

The greater postural deterioration observed in some individuals, as indicated by the quantile and delta analyses, highlights the relevance of individual variability. Accordingly, healthcare professionals should consider supervising initial VR sessions, educating users about possible balance-related symptoms, and, when feasible, performing simple baseline balance screening to support safe and individualized VR implementation.

## Figures and Tables

**Table 1:** Anthropometric characteristics of the participants.

Variables	Values	95% Confidence Interval
Sample size, n (men; women)	28:51	---
Age, years	21.5 (2.5)	21.0; 22.1
Weight, Kg	66.2 (13.9)	63.1; 69.3
Height, m	1.7 (0.1)	1.6; 1.7
Body mass index, Kg/m^2^	23.3 (3.8)	22.4; 24.1

**Table 2:** Impact of 3D virtual reality on postural control.

Variables	3D Virtual Reality		Mean Difference	95% C.I. of the Difference	*p*	Effect Size	Statistical Power (%)
	Off	On					
Frontal sway ^†^, cm	3.5 (3.3)	4.3 (3.2)	0.764	0.577; 0.950	0.001	0.919	99.9
Lateral sway ^†^, cm	2.4 (2.9)	2.6 (2.7)	0.068	−0.086; 0.223	0.383	NS	13.9
Center of pressure ^γ^, cm^2^	2.0 (1.4)	4.2 (4.4)	2.294	1.640; 3.225	0.001	0.849	99.9
Frontal speed ^γ^, cm/s	1.4 (0.5)	1.7 (0.7)	0.315	0.245; 0.390	0.001	0.910	99.9
Lateral speed ^γ^, cm/s	1.2 (0.5)	1.4 (0.6)	0.125	0.075; 0.200	0.001	0.663	98.1

Note: ^γ^ Parametric analyses: values are presented as mean (SD); paired *t*-test; effect size is expressed as Cohen’s d. ^†^ Non-parametric analyses: values are presented as median (IQR); Wilcoxon signed-rank test; effect size is expressed as rank–biserial correlation. NS: non-significant effect.

## Data Availability

The original contributions presented in this study are included in the article and Supplementary Materials. Further inquiries can be directed to the corresponding author.

## Supplementary Materials

The following supporting information can be downloaded at: https://www.mdpi.com/article/10.3390/clinpract16020040/s1. Table S1: Raw data before exclusions and statistical processing.
