# Supplementary material for: Effects of 3D Virtual Reality on Postural Control in Young Adults: Clinical and Practical Implications
Source: Clin Pract. 2026 Feb 13;16(2):40. doi: 10.3390/clinpract16020040 (PMC12939972; doi:10.3390/clinpract16020040)
Supplement: Supplementary file 1 [file clinpract-16-00040-s001.zip › clinpract-4089927-supplementary.pdf]

Table S1. Raw data before exclusions and statistical processing

Fronta sway	Lateral sway	CoP	Frontal speed	Lateral speed	Fronta sway	Lateral sway	CoP	Frontal speed	Lateral speed
1,24	-0,29	1,56	1,33	1,26	1,57	-0,16	6,19	1,53	1,35
1,35	1,85	3,36	1,14	1,05	1,98	1,37	2,56	1,28	1,13
-0,19	-0,63	2,25	1,05	1,01	1,5	0,39	1,63	1,26	0,99
3,15	1,51	2,71	1,12	1,02	3,79	1,94	4,75	1,51	1,07
-0,12	-0,04	1,78	1,16	1,15	0,55	1,28	2,75	1,25	1,17
-1,56	-0,64	2,85	0,96	0,68	-0,28	0,6	7,48	1,46	0,82
-0,38	-2,13	3,56	1,52	1,44	0,6	-1,48	4,4	1,92	1,5
7,53	4,76	4,24	1,39	1,28	7,54	4,61	8,09	1,52	1,42
7,18	4,56	1,57	1,47	1,43	7,28	4,42	4,53	1,74	1,58
0,62	-0,86	5,9	1,18	0,98	-1,14	0,31	6,73	1,68	1,17
-2,17	-0,86	1,71	0,8	0,78	-0,42	0,44	4,9	0,97	0,82
-0,69	-0,57	1,47	1,15	1,04	1,19	-0,03	6,27	1,32	1,12
1,56	-0,71	2,36	1,23	1,24	3,23	-1,23	4,91	1,37	1,26
7,7	5,18	1,48	1,78	1,56	9,04	4,31	3,17	2,31	1,71
8,27	3,53	1,23	1,63	1,45	8,11	4,31	0,94	1,78	1,57
2,1	-1,81	2,11	0,96	0,9	1,24	-1,02	5,02	1,06	0,9
3,01	-0,94	1,49	1,14	1,05	4,27	-0,05	3,21	1,42	1,26
5,68	6,34	0,99	1,19	1,2	6,9	5,8	1,41	1,27	1,19
3,83	5,59	1,06	1,58	1,43	6,09	5,88	4,83	1,81	1,53
5,29	5,44	2,68	1,44	1,26	5,31	5,3	7,02	1,91	1,47
5,21	5,28	2,87	1,33	1,24	6,81	4,56	0,7	1,21	1,12
7,85	4,36	1,56	2,09	1,97	8,23	4,75	7,84	2,61	2,56
7,15	5,25	4,18	2,17	2,21	8,06	5,08	15,86	2,71	2,54
8,87	3,9	1,35	1,45	1,24	8,94	3,7	1,33	2,57	1,82
6,67	5,21	0,83	1,19	1,21	7,49	4,68	1,31	1,26	1,14

7,6	5,15	0,95	1,29	1,26	7,63	5,46	3,03	2,09	1,95
5,7	4,93	7,51	2,28	1,94	6,52	4,6	6,91	2,65	2
7,24	4,57	2,21	1,64	1,8	7,28	4,93	1,83	1,73	1,46
-0,04	-0,54	1,55	1,07	0,98	0,39	-0,37	3,33	1,43	1,08
-1,83	-0,04	1,43	0,96	0,8	-0,77	0,41	3,96	1,15	0,89
2,03	-0,62	3,04	-0,62	0,98	2,92	-0,55	6,38	1,57	0,98
-1,06	0,48	2,54	0,97	0,81	-0,29	1,13	3,15	1,02	0,82
0,69	0,13	0,88	0,92	0,78	0,75	0,23	0,61	1,01	0,8
1,59	0,06	0,96	1,17	1,1	1,91	0,23	4,21	1,42	1,2
4,7	-0,59	2,58	1,52	1,34	4,69	-0,65	2,29	1,64	1,32
4,89	-1,04	2,07	1,4	1,19	5,52	-0,09	2,84	2,04	1,24
1,88	0,2	1,14	1,25	1,19	1,5	0,05	8,2	1,42	1,19
2,65	0,8	1,51	0,85	0,79	4,24	0,85	3,02	1,13	0,86
-0,01	0,08	2,17	1,23	1,06	0,62	0,5	2,04	2,73	1,69
0,46	-0,3	2,49	1	0,74	1,58	-0,44	3,51	1,2	0,79
5,18	4,64	2,8	1,76	1,5	6,29	4,19	2,68	1,64	1,44
7,24	4,99	2,06	1,43	1,35	8,19	5,2	4,79	2,06	2,07
7,16	3,6	2,67	1,71	1,66	7,67	3,43	4,32	2,4	2,13
6,39	5,28	0,94	1,5	1,29	8,5	4,27	3,85	1,72	1,42
7,71	5,86	0,79	2,42	2,31	9,4	4,86	2,29	2,56	2,3
8,04	5,62	1,83	2,48	2,38	7,87	5,83	1,44	2,43	2,36
8,01	4,2	6,35	1,88	1,82	8,57	3,43	15,8	2,31	2,41
2,44	7,8	1,19	1,85	1,76	3,32	8,2	2,21	2,46	2,98
9,04	3,23	1,02	1,53	1,3	8,77	3,95	5,02	1,99	1,71
4,82	6,61	0,84	1,64	1,53	6,3	6,13	1,49	2,02	1,5
5,93	6,4	2,71	1,15	1,03	5,96	6,35	5,72	2,2	1,79
5,85	5,95	1,71	1,5	1,41	6,35	5,75	0,71	1,58	1,43
5,59	5,65	0,81	1,61	1,43	6,08	6,36	2,03	2,15	1,66

7,28	6,11	2,62	1,94	1,72	6,3	6,29	6,61	2,22	2,04
6,37	6,08	0,81	1,61	1,56	7,03	5,14	1,31	1,8	1,59
5,69	5,3	2,64	1,88	1,68	5,39	7,03	3,35	2,3	1,69
-0,96	-1,18	1,76	1,02	1,03	0,68	-0,87	9,66	2,66	1,76
2,37	-0,9	3,06	1,13	0,89	2,06	-0,61	13,78	1,87	1,64
5,7	5,78	1,11	1,93	1,92	6,52	5,39	4,24	1,81	1,59
6,03	5,43	1,17	1,4	1,34	6,42	5,77	3,57	1,65	1,43
4,9	6,21	2,31	1,55	1,52	6,84	4,96	0,97	1,53	1,41
6,63	4,66	2,07	3,65	3,79	6,06	5,98	17,73	2,49	2,36
5,27	5,33	2,34	1,24	0,98	6,45	5,42	2,1	1,35	0,96
6,49	4,77	1,35	1,97	1,81	8,53	3,44	3,15	1,94	1,75
6,98	4,8	2,03	1,94	1,81	9,61	2,73	11,9	1,98	1,99
7,59	4,54	1,06	1,65	1,49	7,79	4,37	1,51	1,95	1,62
2,12	7,4	2,29	1,49	1,45	2,56	7,1	4,07	2,01	1,64
0,61	-1,06	2,63	1,6	1,19	1,84	-0,41	12,23	2,28	1,4
-3,48	-0,25	7,31	1,58	1,42	-2,87	-0,23	9,76	1,52	1,24
0,75	0,13	2,46	1,51	1,25	1,75	0,75	14,28	2,05	1,63
-2,07	1,2	2,77	1,33	1,13	-0,17	1,2	1,72	1,41	1,1
-0,07	-0,85	0,75	0,92	0,72	1,3	-0,56	6,59	1,93	1,05
1,5	-0,1	1,41	1,3	1,24	3,32	-0,8	14,68	1,45	1,32
-1,73	-0,91	6,75	1,41	1,23	0,01	-0,66	20,78	1,7	1,59
2,45	-1,53	5,27	1,82	1,76	3,81	-1,69	16,41	2,63	2,3
-0,06	0,33	4,75	1,17	1,08	-0,45	-0,11	6,33	1,64	1,3
-2,07	0,39	7,36	1,87	1,4	-0,27	-1	19,62	2,66	1,79
0,76	-0,14	4,73	1,48	1,04	0,83	0,73	4,12	1,92	0,86
1,64	-0,91	3,43	1,18	0,91	3,07	-0,95	4,88	1,45	1,01
